# Evaluation of interventions on road traffic injuries in Peru: a qualitative approach

**DOI:** 10.1186/1471-2458-12-71

**Published:** 2012-01-23

**Authors:** Luis Huicho, Taghreed Adam, Edmundo Rosales, Ada Paca-Palao, Luis López, Diego Luna, J Jaime Miranda

**Affiliations:** 1 Facultad de Medicina, Universidad Peruana Cayetano Heredia, Av. Honorio Delgado 430, Lima LI31, Peru; 2 Facultad de Medicina, Universidad Nacional Mayor de San Marcos, Av. Grau 755, Lima LI01, Peru; 3 Instituto Nacional de Salud del Niño, Av. Brazil 600, Lima LI05, Peru; 4 Programa de Investigación en Accidentes de Tránsito (PIAT), Salud Sin Límites Perú, Calle Ugarte y Moscoso 450, Lima LI17, Peru; 5 Alliance for Health Policy and Systems Research, World Health Organization, 20 Avenue Appia, 1211 Geneva, Switzerland; 6 Centro de Trastornos Respiratorios del Sueño (CENTRES), Clínica Anglo Americana, Alfredo Salazar 350, Lima LI27, Peru; 7 Hospital Clínic de Barcelona, Villarroel 170, Barcelona 08036, Spain; 8 Programa Nacional de Empleo Juvenil Jóvenes a la Obra, Ministerio de Trabajo y Promoción del Empleo, Av. Salaverry 655, Lima LI11, Peru; 9 Departamento de Ciencias Sociales y Políticas, Universidad del Pacífico, Av. Salaverry 2020, Lima LI27, Peru; 10 CRONICAS, Centro de Excelencia en Enfermedades Crónicas, Universidad Peruana Cayetano Heredia, Av. Armendáriz 497, Lima LI18, Peru

## Abstract

**Background:**

Evaluation of interventions on road traffic injuries (RTI) going beyond the assessment of impact to include factors underlying success or failure is an important complement to standard impact evaluations. We report here how we used a qualitative approach to assess current interventions implemented to reduce RTIs in Peru.

**Methods:**

We performed in-depth interviews with policymakers and technical officers involved in the implementation of RTI interventions to get their insight on design, implementation and evaluation aspects. We then conducted a workshop with key stakeholders to analyze the results of in-depth interviews, and to further discuss and identify key programmatic considerations when designing and implementing RTI interventions. We finally performed brainstorming sessions to assess potential system-wide effects of a selected intervention (Zero Tolerance), and to identify adaptation and redesign needs for this intervention.

**Results:**

Key programmatic components were consistently identified that should be considered when designing and implementing RTI interventions. They include effective and sustained political commitment and planning; sufficient and sustained budget allocation; training, supervision, monitoring and evaluation of implemented policies; multisectoral participation; and strong governance and accountability. Brainstorming sessions revealed major negative effects of the selected intervention on various system building blocks.

**Conclusions:**

Our approach revealed substantial caveats in current RTI interventions in Peru, and fundamental negative effects on several components of the sectors and systems involved. It also highlighted programmatic issues that should be applied to guarantee an effective implementation and evaluation of these policies. The findings from this study were discussed with key stakeholders for consideration in further designing and planning RTI control interventions in Peru.

## Background

Road traffic injuries (RTIs) are currently one of the leading causes of death at global level. They kill about 1.3 M people annually, with approximately 90% of the deaths occuring in low- and middle-income countries [1].

**Figure 1:**
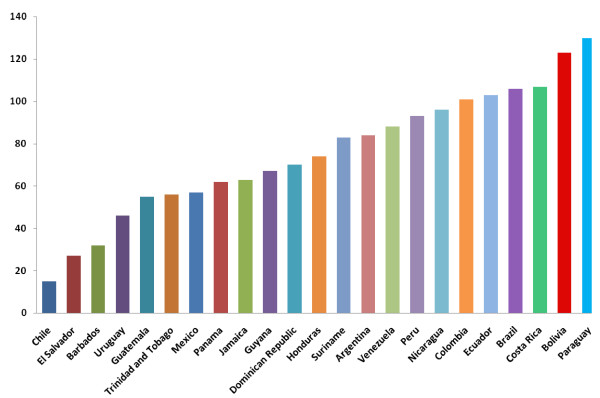
**Ranking of selected Latin American countries by quality of roads**.

Peru is a middle-income country with a sustained migration pace and an increasingly accelerated urbanization process [2]. An integral and comprehensive planning framework has largely been absent in this modernization process, and thus urban growth has resulted in a chaotic landscape, with an insufficient and unfriendly road infrastructure [3-5]. Peru lags behind in quality of roads compared with other Latin American countries [6] (Figure 1). Pedestrians in particular have not been taken into account when designing the cities, and poorly regulated export of used cars has predominated for decades [7]. Therefore, pedestrians and passengers alike have increasingly become exposed to dangerous roads and vehicles [7,8]. In addition, governance and accountability are still weak in the country [9,10], and thus represent a challenge for an effective enforcement of road safety regulations. Not surprisingly then, RTIs have gained increasing visibility in Peru. This scenario is by no means unique to Peru, as other countries in the region and in other regions of the world are also facing similar problems related to migration, urbanization and road infrastructure [11-13]. Thus the lessons learned from the Peruvian experience on RTIs will likely be relevant to other settings as well.

The government and the Peruvian National Institute of Health have considered RTIs a public health problem, and they have recently been ranked as a top priority problem deserving more research and strengthened development of prevention and control public policies [14]. Accordingly, several interventions have been implemented at country and regional level, but there is scarce information about their real impact, let alone about their success and failure factors [15-17], key if policymakers are going to make informed decisions.

**Figure 2:**
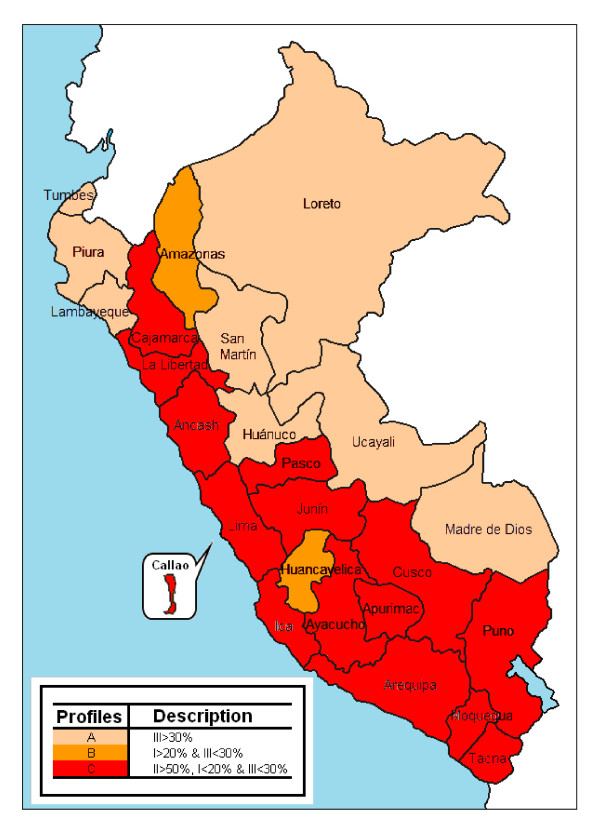
**Profile of road traffic crashes in Peru at departmental level, by type of vehicle involved, 2008**. Groups of vehicles: I: bus, trailer truck, truck; II: automobile, van/station wagon, microbus, pickup; III: Motocar (*mototaxi*), motorcycle; IV, bicycle and tricycle; V: other.

An assessment of the epidemiological profile of RTIs in Peru that we performed, which is reported in more detail elsewhere [18], shows a clear national level increase in road traffic crashes through the study period. The standardized incidence rates in 1973 were 90.4 and 41.6 per 100,000 population for males and females, respectively. In 2008, they were 130.4 and 53.3 per 100,000 population for males and females, respectively. The incidence increased in almost all departments from 1973 to 2008. Of note, at departmental level the incidence increased from 16.2 per 100,000 population in 1973 to 117.5 in 2008 for Ayacucho, and from 34.0 in 1973 to 192.2 in 2008 for Ucayali, whereas it varied from 120.0 to 311.0 in Lima for the same period. In 2008, the proportion of families living below the poverty line represented 68.3% in Ayacucho, 45.0% in Ucayali, and 19.4% in Lima [19]. This clearly shows a disproportionately higher increase of road traffic crashes in poorer inner cities compared with Lima, the capital city. Moreover, while automobiles, buses, vans, and pickups were involved in about 70% of all road traffic crashes at national level in 2008, 3-wheelers motorcars or *moto-taxis *were consistently the most frequently involved vehicles in rainforest departments (Loreto, Madre de Dios, and Ucayali), accounting for more than two thirds of total events in those areas (Figure 2). *Moto-taxis *are fragile and hazardous small vehicles [1], and they are widely used in inner cities of Peru for public transportation of up to three persons.

As for RTI figures by type of road users, we could obtain detailed data at Lima level for 2006 [18]. This information showed that 44% of victims were pedestrians and passengers, while 54% were drivers of motor vehicles. About 72% of victims were between 5 and 45 years old, and were mostly males (76%). For people aged 0 through 19 years old, pedestrians and passengers accounted for the greatest proportion of victims, while drivers predominated in the 20-59 years old group.

Policy interventions for reducing RTIs and their consequences are complex in nature; they are implemented in diverse and ever changing settings, and invariably trigger different reactions and changes in the diverse systems involved in their implementation [20,21].

The objective of this study was to evaluate current interventions implemented to reduce RTIs in Peru, and to identify key programmatic aspects in the process of design, implementation, monitoring and evaluation of RTI interventions that may be useful at global and local level for making more informed policy decisions, so as to increase the likelihood of successful implementation of such interventions.

## Methods

### Qualitative evaluation of RTI interventions

Qualitative studies focused on policy makers and implementers may allow understanding of underlying success or failure factors operating during the planning and implementation phases. We therefore performed a qualitative study for evaluating various design and implementation aspects of different RTI interventions in Peru, and to gain additional insight from interviewees about redesign feasibility of the evaluated interventions. We finally included brainstorming sessions as part of a systems thinking approach [22], to reveal positive and negative effects of RTI interventions on the different involved systems and on their diverse components.

First we performed a qualitative study of the different RTI policy interventions that have been implemented in Peru within the last 5 years at national and regional level, and that were aimed at prevention and control of road traffic crashes. We specifically performed in-depth interviews with policymakers and technical officers responsible for various RTI interventions in three cities (Lima, Ayacucho, and Pucallpa) representing three different geographical, socioeconomic and cultural regions of the country. These are the coast, the Andes and the rainforest, respectively. National level interventions have their central managerial headquarters in Lima. This was one reason why we chose Lima as one of the study settings. The other reason was that it accounts for the greatest proportion of RTIs in the country. We chose to perform in-depth interviews rather than focus groups because we wanted to avoid that hierarchal and power structures could result in less frank and less productive discussions among policymakers and technical officers. Additionally, bringing together these participants for group discussions were difficult due to their different and busy work schedules.

The main objective of the interviews was to gain a deeper understanding of the diverse aspects of design, implementation, monitoring and evaluation of RTI interventions currently being implemented, in order to use them as programmatic inputs in the process of designing new RTI interventions or when considering redesign options. Interview guides were applied. Specific thematic issues considered for assembling the interview guides included: a) general programmatic characteristics of interventions; b) operational aspects of interventions; c) priority RTI risk factors addressed by interventions; d) neglected RTI risk factors; e) success and failure factors; e) achievements, limitations, and lessons learned during implementation.

Qualified researchers performed the interviews. The core research team composition included a mix of two epidemiologists, a medical doctor, a sociologist, two economists, and two psychologists, all with expertise in qualitative studies and familiar with the three study settings. They are part of a national comprehensive research program on RTIs supported by the Peruvian National Institute of Health, which has also performed other qualitative studies on diverse aspects of RTIs. Several preparatory group sessions were additionally performed for further highlighting the quality standards required for conducting qualitative studies. In addition, the field interviewers were selected within each city on the basis of previous documented experience in performance of in-depth interviews and their knowledge of local geography and cultural characteristics. Furthermore, pilots were performed in each setting for refining the study tools and for anticipating potential problems during the field phase. During the interviews participants were allowed to develop their discourse freely on each theme raised, and prompters were used on an as-needed basis.

We adopted the grounded theory approach [22,23], and performed consequently a purposive sampling, aiming at getting the most relevant information by approaching policymakers and technical officers familiar with the design and implementation phases of RTIs interventions. Therefore the individuals were interviewed until the theoretical saturation point was reached, that is the information provided did not contribute to further refinement of our theoretical framework [22].

Participants were approached in their work setting, and arrangements were made to avoid as far as possible disruption of their duties, scheduling the interviews out of their work hours. Participants signed informed and voluntary written consent forms. Participation was entirely voluntary. All interviews were recorded but identities of participants were preserved. Each interview lasted from 45 to 60 minutes. Notes were taken during the interviews, and were used afterwards to produce a complete transcript of each interview in Spanish.

A total of 19 in-depth interviews were performed to 8 managers/policymakers and 11 technical officers responsible for RTI interventions in Lima, Ayacucho and Pucallpa (Ucayali). They identified and commented on 17 RTI interventions that are currently being implemented in Peru. Table 1 shows a summary of these interventions and their key characteristics.

**Table 1:** Major RTI interventions in Peru assessed through in-depth interviews

Intervention	Main intervention (content)	Implementer	Declared target group	Actual coverage	Existing M&E system	Main challenge for effective implementation
Zero Tolerance	Compulsory technical review of vehicles at departure point and driver alcohol screening	Ministry of Transport	National	Lima and a few other cities	No	Limited logistics and ineffective enforcement

Strategic plan on road safety	A comprehensive set of road safety interventions to be implemented during 2007-2011 period	Various ministries	National	Scattered and limited implementation	No	Non-binding technical plan, without effective political commitment for actual implementation

Educational Program on road safety	Training of primary and secondary teachers on road safety educational messages	Ministry of Education	National	Unknown	Unavailable	Disregard of evidence showing lack of impact of isolated education interventions

Alcohol-impaired driving program	Random use of breath-testing devices at different road points	Police	National	Unknown	No	Limited logistics and ineffective enforcement

Touring school on road safety	Educational campaigns for 8-12 years children	Not-for profit private sector	Regional (Lima and Callao)	Unknown	Unavailable	Disregard of evidence showing lack of impact of isolated education interventions

Local educational programs on road safety	Educational campaigns for different target groups	Regional and local governments	District and province level across the country	Unknown	No	Disregard of evidence showing lack of impact of isolated education interventions

Local traffic planning programs	Varying set of regulations for limiting traffic of motor vehicles	Regional and local governments	District and province level across the country	Unknown	No	Lack of clear objectives and goals during planning and implementation phases

**M&E: **Monitoring and evaluation

Our theoretical framework underlying the qualitative study was based on the reflexivity principle [24], and therefore took into account our background position. The framework assumed that RTI interventions had been implemented with limited success in Peru for various reasons. These reasons were inferred from a desk review performed for exploring documents related to implemented interventions, and after contrasting their characteristics with the recommendations of the World Health Organization (WHO) how to design and implement RTI interventions [1]. The first assumption was that the implemented interventions had not been evidence-based and thus they had been excessively centered on isolated educational interventions. The second assumption was the absence of a systematic planning when selecting the candidate interventions. The third assumption was that epidemiological profile of RTIs at local level and socioeconomic and cultural contextual factors were largely neglected when selecting and planning implementation of RTI interventions. Fourth, it was assumed that implementation was most frequently scattered or with limited coverage to result in a measurable impact. Fifth, it was assumed that the interventions had not received actual and effective support from the highest political levels. The sixth assumption was a limited community involvement in the design and implementation of RTI interventions, which reduced the likelihood of an effective enforcement of regulatory interventions such as speed limits. Finally, it was considered that governance and accountability levels are perceived by community members as too weak at central and local levels, which also reduced the chances of effective enforcement of potentially useful interventions such as speed limits, seat-belt use, or prevention of alcohol-impaired driving.

In accordance with the above described theory framework, a number of a priori thematic categories were established as the analysis starting point. The proposed analysis categories included: a) driving force(s) for selecting RTI interventions; b) main programmatic characteristics of the interventions, encompassing the whole policy process, from the design phase through to impact measurement; and c) monitoring and evaluation activities.

The analysis plan included transcript of all recordings to Microsoft Word computer software. Then, based on the preliminary thematic categories, a critical reading of a small number of transcripts was performed, to identify initial categories and to construct a book of codes. Coding of interviews was accomplished by using Atlas.ti (Scientific Software Development GmbH; Berlin, Germany). Building on the emerging categories and following our grounded theory model, iterative discussions among researchers were performed, until the point of saturation was reached, and no new codes or thematic issues emerged. Both core researchers and field staff were involved in the analytical discussions through reflexivity and triangulation [24], to broaden the analysis perspectives.

### Stakeholders meeting

We then held a meeting with stakeholders representing in a balanced way the different sectors involved in planning or implementation of road safety interventions in the country, as recommended [25,26]. They included representatives of the Technical Secretariat of Road Safety Council, Lima Municipality, the non-governmental organization (NGO) Luz Ámbar, the Peruvian National Institute of Health, the National Policy of Peru, and the National Sanitary Strategy of Road Traffic Crashes. The aim of the meeting was to present the results of the in-depth interviews mentioned in the previous paragraph, and to stimulate a new round of discussions about key programmatic considerations to take into account when planning the design and implementation of RTI interventions in the country.

Semi-structured questions were used as prompters to guide the discussion on specific issues related to the design, implementation and evaluation of current RTI policy interventions. This was followed by a free discussion that ended up with the elaboration of a consensus summary.

### Brainstorming sessions with stakeholders

Additionally, a role-play brainstorming session was organized with a group of participants representing key policymakers and implementers from different sectors. This was complemented by email discussions between them, moderated by one of the authors (LH). The objective was to solicit their perceptions on the potential system-wide effects of a selected intervention (Zero Tolerance), and to subsequently identify adaptation and redesign needs for this intervention [21]. Zero Tolerance is a compulsory inspection aimed at public inter-provincial buses at the points of departure. It requires that drivers hold a professional ad-hoc driving license, a certificate attesting that the bus is operational, a successful technical inspection of the bus, a valid compulsory insurance against road traffic crashes, and a negative alcohol testing for drivers. The implementation of Zero tolerance is led by The Ministry of Transport with participation of the Police and the Ministry of Justice that should lead to enforcement of corrective measures resulting from the inspections.

## Results and discussion

### Qualitative evaluation of RTI interventions

Based on our preconceptions (reflexivity principle) and on the iterative discussions performed until the theoretical saturation point was reached, the analysis issues were consolidated eventually into three main categories, namely driving forces for selection of RTI interventions, programmatic characteristics of interventions, and monitoring and evaluation aspects.

A common concern expressed by most interviewees was the limited scale of implementation of RTI interventions. Despite initial plans for implementation at scale across the whole country, an overwhelming number of these interventions are implemented only in few major cities, and in a very scattered way (Table 1), reducing therefore their potential impact. A noticeable example is Zero Tolerance, considered later in this report.

#### Selecting RTI interventions in Peru

There was a general consensus that the main motivation for selecting RTI interventions varied in different settings, although a salient driving force highlighted was the pressure exerted by the media and the community. This finding is in line with our assumption that interventions are not chosen on the basis of existing evidence, but also reveals additional driving forces, most notably the media influence, which has also been reported in other settings [27]. As an interviewee explained:

*Every day we read or watch news on crashes and victims. Most frequently drivers are shown as those who cause them. The media forces politicians to choose quickly any intervention, no matter if it is effective or not, because they fear the media and also the community anger*.

(Officer/Implementer, Technical Secretariat of Road Safety Council, Lima)

Drivers are consistently publicized as those with the greatest responsibility in the occurrence of road traffic crashes, being perceived as actors with deviant behaviours, and thus the interventions targeted to this group occupy a prominent place at all levels in the country. The media also promotes widely education interventions; an approach that has therefore received prominent attention by policymakers, although the evidence questions its effectiveness, especially when implemented in isolation [1]. An interviewee put it this way:

*We all know that most drivers have inadequate behaviours and psychological problems, and that they cause the crashes. Thus we would like to change those behaviours, we would like to educate them*.

(Technical officer of Road Safety Education, Lima)

Interestingly, the interviewees agreed with the media on the importance of strengthening current students and drivers' education interventions despite informing them about available evidence from Peru suggesting otherwise [27]. This indicates that it is not enough to ensure that research information is shared. It is more important that it is packaged appropriately and specifically for use by policymakers and that policymakers are equipped with the necessary skills to discuss and evaluate the quality of evidence they are presented with [28].

Another salient factor influencing the selection of RTI interventions was the availability of refundable external funds conditioning the loans to the development of road safety strategies, suggesting the need to strengthen national and local initiatives aimed at identifying the priority aspects related to RTI interventions, while also taking advantage of external sources of funding and policy influence. One example of such an external driving force is a World Bank loan in the 1990s that required the Peruvian government to assign 10% of the loan to the development of a comprehensive road safety strategy. As a policymaker explained:

*The World Bank gave money to our country when Fujimori was president, and said that they were going to borrow us 100 M bucks, but that government should spend them in road safety. Thus a consultancy was commissioned, and this concluded that the country needed a cross-sectoral road safety organization. And thus the National Council of Road Safety was created in 2004*.

(Policymaker, Technical Secretariat of Road Safety Council, Lima)

At regional level, the need to comply with municipal or regional level mandates; efforts by NGOs focusing on road safety; and the decentralization process at regional and local governments were all considered as important driving forces. They illustrate the need of considering context-specific aspects when planning and implementing RTI interventions at sub-national level.

#### Main programmatic drawbacks related to RTI interventions

Interviewees consistently identified programmatic drawbacks in the policy process of design and implementation of RTI interventions in Peru, which include:

(a) Lack of clear and sustained political and budgetary support for both national and local level interventions, which are aggravated by frequent change of high-level policymakers. As an interviewee explained:

*"...political will has been absent in all the efforts. We always notice a lack of interest. Whenever a new minister is in charge, which happens all the time, he has to be informed again and again, and we have to wait for him to become familiar with the problem*.

(Coordinator, Violence Observatory, Universidad Peruana Cayetano Heredia, Lima)

(b) Lack of clear planning and implementation guides, which lead to a high degree of improvisation when selecting potential RTI interventions, and when implementing them. An interviewee put it this way:

*"...not only for traffic injuries, we need to made and effort of thinking on all things that happen in our country. But today things are done without thinking. We spend a lot of money implementing things without planning them carefully, without measuring their impact. Then they are abandoned and no one knows why they didn't work. And we do not learn from our mistakes*.

(Coordinator, Violence Observatory, Universidad Peruana Cayetano Heredia, Lima)

(c) Lack of regular, planned training and supervision activities; and scarcity of dedicated staff to implement and enforce RTI interventions. Policymakers and implementers felt that besides structural and budgetary limitations, scarcity of trained human resources and of a supportive supervision system limit the implementation of RTI interventions. One technical officer explained:

*With the problem of road traffic injuries, we have structural and budgetary limitations, and limited human resources, but also insufficient training and supervision. We don't even have ad-hoc physical spaces for performing training activities*.

(Technical officer, Training Unit, Municipality of Pucallpa, Ucayali)

(d) Ineffective coordination between the different sectors involved, which is particularly important when designing and implementing complex, crosscutting RTI interventions. A policymaker put it this way:

*Definitely, if you don't act in a multisectoral way, you will fail. The safety road issue needs cooperation, coordination, if you want to have an impact*.

(Central level policymaker, National Strategy for Road Traffic Injuries Ministry of Health, Lima)

(e) Insufficient community participation, which reveal the need to consider more seriously the opinions and the active participation of community members when planning and implementing the interventions. This perception is line with our anticipated theoretical framework, and reduces the likelihood of an effective enforcement of several regulatory interventions. As a policymaker explained:

*Very few interventions in our country consider actually community participation. Fortunately, our education program for road safety includes community members that are very committed to contribute. They are very important because they talk to their fellow citizens, and they are influential*.

(Responsible, Road Safety Program, San Borja Municipality, Lima)

(f) Lack a reliable and fully functional information system. This limits seriously the assessment of the actual burden posed by RTIs and the possibility of measuring reliably the impact of RTI interventions. As an interviewee explained:

*We need to work with clear objectives, we need to have a good database on road traffic crashes... we need those indicators to measure our work*.

(Responsible, Road Safety Program, San Borja Municipality, Lima)

These perceptions were broadly in agreement with our theory framework, although interviews revealed further context-specific aspects not anticipated, such as lack of adequate regulations for local transport vehicles.

At regional and local level, the most important programmatic issue raised was weak regulatory framework and enforcement of regulations. In Ayacucho and Pucallpa in particular, interviewees were concerned about the lack of adequate road safety regulations to address the continuous increase of motorcars or *moto-taxis*, with additional emphasis expressed on the limited success in the enforcement of the existing regulations. As local policymakers explained:

*I think that we need a tight control. We really need that the police make an effective control of road safety infractions... We have too many regulations, but they are not well planned. And they are not enforced, you know. People don't care about those regulations*.

(Responsible, Training Unit, Municipality of Pucallpa, Ucayali)

*"...There are too many moto-taxis. And their drivers don't care about road safety regulations. But we don't have good regulations for them. They are fragile and dangerous as well. We also need to educate drivers of moto-taxis*.

(Responsible, Training Program for moto-taxis, Coronel Portillo Municipality, Pucallpa)

With regard to suggested actions to improve the implementation of current RTI interventions, the most important ones were the need for a stronger multisectoral coordination of scaling-up activities; and the need to develop strategies to overcome the cultural, social and geographical diversity of the country, and to address the specificities and modalities for enforcing regulations in the different regions. As a local policymaker explained:

*...well, the best lesson learned is the need of coordinated work: authorities, transport colleagues, all actors. Then we can have more efficient actions, better results. But we should remember that each place has its own problems, moto-taxis for instance, are a problem here, but not so much in Lima. Also, you know that use of seat belts are going to be very difficult in Ucayali, because it's so hot here. And can you imagine seat belts in moto-taxis? Really hard*.

(Policymaker, Road Safety Training Unit, Ministry of Transport, Ucayali)

Regarding the media influence at local level, as the following quote highlights, a wide but responsible participation and support from the media was also identified as an important and crucial step in the successful implementation of RTI interventions and their enforcement:

*Well, journalists are very important, but they shouldn't be so sensationalists, they should be more responsible when they release the news. Only in that way they are going to help really to improve the problem of road crashes*.

(Policymaker, National Strategy for Road Traffic Injuries Ministry of Health, Lima)

#### Monitoring and evaluation

Finally, the interviewees consistently pointed out that none of the interventions have systematic monitoring and evaluation activities, and no clearly identified process and impact indicators to evaluate progress along the way. When they exist, they are most often related to the measurement of administrative goals, rather than to the impact of the implemented interventions themselves or to identification of aspects needing improvement. This reveals that monitoring and evaluation limitations are far more dramatic than we had assumed in our theoretical framework:

*...no, we don't monitor, we don't have the monitoring tools, we don't have evaluation indicators, we don't have the necessary budget*.

(Policymaker, Communication Unit, Road Safety Decentralization Program, Lima)

*It is hard to talk about monitoring, you know, especially when implementation of activities is still limited. We have explored this, however, and facilitators should be trained, so they can monitor and offer counseling*.

(Policymaker, Safety Road Education Program, Ministry of Education, Lima)

*Informal evaluations... yes, we do. But actual evaluation of results and monitoring, no, because it is not sustainable. Probably in the future, after massive training of drivers*.

(Policymaker, Training Courses Section, Coronel Portillo Municipality, Pucallpa, Ucayali)

*We have a supervisor who tries to ensure a small evaluation at the end. We were thinking to give an incentive to schools that show improvement in road safety. We would like to perform a theater representation*.

(Implementer, Safety Road Project, Ayacucho Municipality)

Although they acknowledged the existence of various information systems in the police, Ministry of Transport, Ministry of Health, and the private sector, which could be used as useful information sources for monitoring and evaluation efforts, participants expressed concern about the quality and completeness of the assembled information, the limited access to the existing information, and the lack of coordination and integration of these different information systems. This finding, which we did not specifically include in our theoretical framework, highlights that a construction of a functional and integrated information system for RTIs remains a pending task at national and local level:

*...we have agreed to share the information. The police, the judicial system, the insurance companies and other sectors have agreed. But there are no concrete results. For example, we wanted to use the district police information, but they didn't want. They said that there was not superior approval. We need written agreements, maybe a law. We need to improve the quality of the information. It needs to be complete. If there are not data, we don't have monitoring and evaluation, you know*.

(Policymaker, Health District, Lima)

*"...we have just started a joint work with the statistics sections of diverse sectors through the regional government. A Coordination Committee has been established to allow sharing of information on violence and road traffic crashes. We have met to coordinate. We hope to have access to different information sources*.

(Chief, National Police, Ayacucho)

### Stakeholders meeting

The meeting generated a wide array of ideas and suggestions applicable to the design, implementation and evaluation phases of the policy process. Many of them were consistent with those provided in the in-depth interviews presented above. The most important or recurring ones are highlighted here.

First, the participants emphasized the need to promote intersectoral alliances both for normative and operational aspects of RTI interventions, as otherwise overlapping, and sometimes-competing efforts will hamper the impact and effectiveness of different interventions.

Second, they insisted on the need to strengthen enforcement of road safety regulatory strategies. In particular, they emphasized that the police needs to be supported from inside and outside to improve its social surveillance role. This would include strengthening of all aspects of the police system, including audit and accountability mechanisms, training, logistic and budgetary aspects. The lack of availability of even basic supplies such as alcohol measurement devices and of combustible fuel for the patrols were highlighted as dramatic examples that jeopardize an effective participation of police in the implementation of RTI interventions such as control of speed limits and alcohol-impaired driving.

Third, the participants highlighted the importance of sustaining and improving technical training and regular supervision of human resources involved in different aspects of RTI implementation efforts, at both national and local level.

Fourth, they felt that improvement of quality and accessibility of information related to RTIs is a pending accomplishment. Although several information systems are in place, there is a critical need for a thorough process to improve the structural and functional aspects of each information system, to identify core indicators, to have easy access to meaningful data, and to effectively coordinate and integrate the generated data to get reliable and updated epidemiologic profiles of RTIs at national and sub-national level.

#### System-wide effects of zero tolerance

The brainstorming session and email discussions proved to be a very instructive and useful exercise that can be easily repeated for other interventions. The output of this exercise for Zero Tolerance is summarized in Table 2. Zero Tolerance inspections are currently limited to main departure points in Lima and a few large cities of the country. Buses and drivers undergoing inspection are not chosen randomly or systematically. While inspection of vehicles and drivers should occur with the joint participation of Ministry of Transport officers, police officers and a public prosecutor, in reality, they are performed sporadically by a Ministry of Transport officer, who is often short of the necessary equipment and public support for an effective enforcement of the corrective measures. Early in its implementation, unrealistic expectations for the potential impact of Zero Tolerance were raised both by the media and politicians. It was burdened, therefore, with ample discredit and wide public criticism that resulted in decreased political commitment and limited allocation of resources. The most important perceived negative system-wide effects were unplanned diversion of human resources and unfulfilled aims to setup a functional information system for monitoring and evaluation. Important potential positive effects such as empowerment of passengers and accountability of public bus drivers were only partially accomplished (Table 2).

**Table 2:** Prioritized perceived system-wide effects of Zero Tolerance

Importance given 1 = high, 5 = low	Effect	Positive + or Negative -	Likelihood (high, medium, low)	Importance (high, medium, low)	Sector/Sub-system involved
1	Narrow target group therefore low impact (inter-provincial public buses only)	-	High	High	All sectors-All building blocks

1	Decreased political commitment due to wide criticism	-	High	High	Central and local government-Leadership and Governance

1	Decreased budget and logistic resources due to criticism	-	High	High	Central and local government-Financing

1	Diverted HR and understaffing in involved sectors (MoT, Police, Ministry of Justice)	-	High	High	Crosscutting-HR & Delivery

1	Decreased police & passengers support to MoT officers	-	High	High	Crosscutting-Delivery

1	Increased accountability perception	+	Low	High	Drivers and vehicle owners-Governance and accountability

1	Opportunity for passengers empowerment	+	Medium	High	People-Governance and accountability

1	Unfulfilled aim to setup information system for M&E	-	High	High	MoT and Police-Information

2	Overburdened police workforce	-	High	High	Police-Delivery

2	Opportunity for coordinated crosscutting activities	+	Medium	High	All sectors-Delivery

3	Unrealistic media-driven expectations	-	Medium	Medium	Media-Leadership & Governance

3	Discrimination feeling of private transport sector (the "bad guys")	-	High	Medium	Private transport sector-All building blocks

4	Overlapping of police and MoT officers' activities	-	Medium	Low	MoT & Police-All building blocks

4	Traffic crowding at check points	-	Medium	Low	Crosscutting-Delivery

MoT: Ministry of Transport; HR: Human resources

Overall, the brainstorming exercise revealed that although there is room for substantial redesign of Zero Tolerance at different building blocks of the health system and the different sectors involved, policymakers may also wish to reconsider whether it deserves continued implementation, due to its very narrow target group (only public inter-provincial buses), and thus the remote likelihood of any measurable impact on the overall rate of RTIs.

### Considerations for a revised RTI interventions plan using a systems approach

#### General lessons for successful implementation of policies

As a result of the different activities and findings described above, supplemented with additional relevant literature [29-32], we identified a list of key programmatic components that we believe are critical for the successful implementation of any policy intervention, including but not limited to RTIs:

• Effective and sustained political commitment

• Comprehensive, evidence-informed planning phase through a wide consultation process

• Defined and sustained budget line

• Adequate initial and ongoing training of human resources

• Effective and regular supportive supervision

• Effective and functioning monitoring and evaluation system in place

• Effective and functioning information system

• Strong governance and accountability mechanisms at national and local level

#### Programmatic considerations related to RTI interventions

The qualitative approach we used for appraising the RTI problem revealed key programmatic aspects that should be taken into consideration when designing any RTI policy intervention. First, candidate interventions should be identified and selected through a wide consultation process with stakeholders representing all relevant actors, and after careful appraisal of the scientific evidence. Then they should be carefully designed, with defined objectives, target groups, as well as inputs, outputs, outcomes and impact indicators to be assessed through a functioning monitoring and evaluation system [32]. A summary of key recommendations emerging from this process for selecting and planning RTI policies follows:

A. Context

• Consider current and future trends of migration from inner cities to capital city and from rural to urban areas across the country

• Consider current and future needs for urbanization planning in cities to avoid increase of chaotic and unsafe roads

• Consider needs for improving quantity and quality of road network

• Consider influence of regulations on importing unsafe motor vehicles on the impact of RTI interventions

B. Systems thinking

• Use a wide consultation process that includes effective community participation to incorporate the views of all relevant actors

• Consider efforts of strengthening policy and judiciary systems as critical elements to effectively enforce road safety regulations

• Explore intended and unintended consequences of proposed RTI interventions (for instance, prohibition of old buses or *moto-taxis *may result in social unrest and increase of unemployment rate, particularly in inner cities)

C. Evidence-based interventions

• Discourage isolated educational interventions as they have been shown to be ineffective

• Promote bundle interventions that include enforcement strategies, as they are likely to be the most cost-effective interventions

• Consider that combinations of interventions will be different in different sub-national settings (for instance, emphasis on *moto-taxis *in rainforest cities)

D. Impact evaluation

• Develop a clear conceptual framework considering design, implementation, monitoring and evaluation steps when planning any RTI intervention

• Define in advance clear effects and impact indicators and put in place a process to collect them

• Avoid undue emphasis on RCT paradigm, due to its practical and ethical limitations, and its inability for answering questions beyond what works or not

It is reassuring that our proposed general and RTI-specific programmatic recommendations are generally in agreement with various experiences in other low- and middle-income settings with regard to identifying and applying key aspects for successful implementation of RTI interventions, but considering also context factors such as migration trends, urbanization planning, quality of road network, and vehicles importing regulations. Take for instance the experience of Curitiba in Brazil, or of Bogota, the capital city of Colombia. A Bus Rapid Transit (BRT) system combined with a long-term land use planning was established in Curitiba since 1965 as part of a Master Plan to convert it in a livable city calling for a cultural, social and economic transformation of the city [33]. A traveller survey was performed in1991, showing a reduction of about 27 M auto trips per year, 28 percent reduction of particular car users, use of about 30 percent less fuel than other Brazilian cities of its size, resulting in one of the lowest rates of ambient air pollution in the country. The Master Plan has been implemented along with other road safety measures comprising legislation to impose stiff penalties, media coverage of the new regime, and better enforcement, which is associated to at least 25% reduction in traffic fatalities [34]. Currently Curitiba BRT serves more than 1.3 M passengers, about 50 times the number from 20 years ago. Eighty percent of travelers use the express or direct bus services, and citizens spend only about 10 percent of their income on travel, much below the national average.

In Bogota, a road safety programme combining land use and transport measures was implemented since 1995, with careful planning, the necessary budget allocation, and with an active and sustained involvement of central and local governments and of the community [35]. The emphasis of the programme was put on meeting the needs of Bogota's non-motorized road users and to improve public transport. Measures included building cycling and pedestrians-only routes that included a car-free route, banning cars circulation in the city centre at peak times, and implementing a high-capacity metropolitan bus system that costed US$ 300 M and was able to transport 700 000 people a day. This bundle programme led to a 50% drop in traffic-related fatalities between 1995 and 2002 [36].

Building on Brazil and Colombia experiences, a programme of provision of a rapid massive bus transport system was implemented in Lagos (Nigeria) in 2008, adapted to the local context, together with other road safety regulatory enforcement measures and investment in infrastructure needs for an enabling environment, with the critical role of commitment and leadership at the highest political levels as an essential success ingredient [37]. Although an impact evaluation on reduction of road traffic injuries is still pending, this programme has already shown an increase in cheaper, safe, comfortable and efficient transportation for over 200 000 commuters, as well as a 40 percent reduction in journey time and a 35 percent reduction in passengers waiting time.

Another illustrative example comes from Thailand, where strong political leadership, responsibility delegation as part of the decentralization process, use of information from RTI databases, positive media influence and multisectoral participation (including community groups, NGOs, and advocacy groups), allowed the development of a comprehensive RTI prevention policy [38]. Interestingly, while the provinces themselves were responsible for preparing the provincial level plans for action, they were developed by local multisectoral public agencies, and at the same time they were largely in agreement with national goals and objectives. The plans cover law enforcement, public education, traffic engineering, emergency services and information systems. Although initially financed on an ad-hoc basis, by the end of 2003 a regular budget was dedicated to road safety. This RTI prevention policy was launched in Thailand building on lessons learned from previous efforts that had not led to measurable impact on RTIs, because they had disregarded various programmatic aspects that were only incorporated in the current initiative [38].

Likewise, it is reassuring that the main programmatic issues raised by participants in our study are well in line with those recommended by the WHO for a better planning, implementation and evaluation of RTI interventions [39]. The Make Roads Safe Campaign of the Commission for Global Road Safety has also defined the programmatic building blocks for a concerted global effort to reduce the rising toll of road deaths and injury [40]: international commitment to a decade of action with global targets, strengthening of national planning adopting a safe systems approach, mobilization of additional financing, integration of road safety into road design, and delivery of road safety "vaccines" putting vulnerable road users first.

At the same time, our results also highlight particular, context-specific issues that our national and local policymakers should consider when selecting RTI interventions, such as the need to think on alternative public transport vehicles, other than *moto-taxis*, which are too fragile and hazardous, and alternatives other than sub-standard quality vans and microbuses. It is encouraging that very recently various local governments (municipalities) have pushed forward a renewed impetus to interventions addressing those specificities. Examples include a) the prompt establishment of a Bus Renewal Scheme ("Bono de Chatarreo" in Spanish) that allows taxi drivers to discard their old and pollutant cars and get in return a cash amount as partial budget for buying a new car; b) the enforcement of a new regulation (Model Bus or "Bus Patrón") that makes compulsory the massive change of a majority of public buses that do not comply with standard safety and environmental standards by other, internationally certified vehicles; and c) an increased pressure of most segments of the civil society to parliament for passing a law prohibiting the import of old pollutant cars, most of which are currently used for taxi service. These specificities, similarly to the key programmatic issues raised by the interviewees, are also mentioned by WHO programmatic recommendations [40], as key policy aspects to take into account at national and local levels.

## Conclusions

In this paper we described the process and the utility of using a systems approach to address policy questions related to RTIs. Strengthening of key programmatic aspects of the different interventions and systematic consideration of potential system-wide positive and negative effects are crucial through the whole policy process, from design through to implementation and evaluation phases. Without involvement of key stakeholders from all sectors involved, it is unlikely that any of these interventions will reach an impact, since coordination and collaboration between sectors are crucial to the successful implementation and enforcement of chosen strategies.

Lessons learnt from this process were summarized in a series of key programmatic aspects and principles for consideration in reviewing and revising the existing RTIs policy interventions plan. While this is as far as we can go with this process, we hope that the findings from this study, and more importantly the process itself that was richly informed and strengthened by the involvement of stakeholders, will help moving this agenda forward. We particularly hope that key stakeholders involved in the design and implementation of RTI policies in Peru will be inclined to take the evidence generated from this study several steps forward, to increase the likelihood of successful implementation of these interventions, and eventually to reach a measurable impact at population level.

## Competing interests

The authors declare that they have no competing interests.

## Authors' contributions

LH conceived the paper and wrote the first draft and the final version of the manuscript. All other authors made substantial contributions to the content of the final version. All authors read and approved the final manuscript.

## PIAT working Group

Members of the PIAT (Programa de Investigacion en Accidentes de Transito) Working Group: Luis Huicho, J. Jaime Miranda, Ada Paca Palao, Edmundo Rosales, Luis López, Diego Luna, Pablo Best (Universidad Peruana Cayetano Heredia, Lima, Peru), Miriam Egusquiza (Salud Sin Límites Perú, Lima, Peru), Claudia Lema (Salud Sin Límites Perú, Lima, Peru), and Esperanza Ludeña (Salud Sin Límites Perú, Lima, Peru).

## Pre-publication history

The pre-publication history for this paper can be accessed here:

http://www.biomedcentral.com/1471-2458/12/71/prepub
